# Lack of evidence for a fine‐scale magnetic map sense for fall migratory Eastern North American monarch butterflies (*Danaus plexippus*)

**DOI:** 10.1002/ece3.9498

**Published:** 2022-11-16

**Authors:** Patrick A. Guerra, Adam F. Parlin, Stephen F. Matter

**Affiliations:** ^1^ Department of Biological Sciences University of Cincinnati Cincinnati Ohio USA; ^2^ Department of Environmental Biology, College of Environmental Science and Forestry State University of New York Syracuse New York USA

**Keywords:** flight orientation, geomagnetic signposts, magnetic declination angle, magnetic inclination angle, migration, navigation, overwintering

## Abstract

How first‐time animal migrants find specific destinations remains an intriguing ecological question. Migratory marine species use geomagnetic map cues acquired as juveniles to aide long‐distance migration, but less is known for long‐distance migrants in other taxa. We test the hypothesis that naïve Eastern North American fall migratory monarch butterflies (*Danaus plexippus*), a species that possesses a magnetic sense, locate their overwintering sites in Central Mexico using inherited geomagnetic map cues. We examined whether overwintering locations and the abundance of monarchs changed with the natural shift of Earth's magnetic field from 2004 to 2018. We found that migratory monarchs continued to overwinter at established sites in similar abundance despite significant shifts in the geomagnetic field, which is inconsistent with monarchs using fine‐scale geomagnetic map cues to find overwintering sites. It is more likely that monarchs use geomagnetic cues to assess migratory direction rather than location and use other cues to locate overwintering sites.

## Graphical abstract

Secular shifts in the Earth's geomagnetic field combined with a lack of change in abundance in the overwintering habitats of the migratory monarch butterfly indicate that it does not use an inherited geomagnetic map sense to locate overwintering sites.

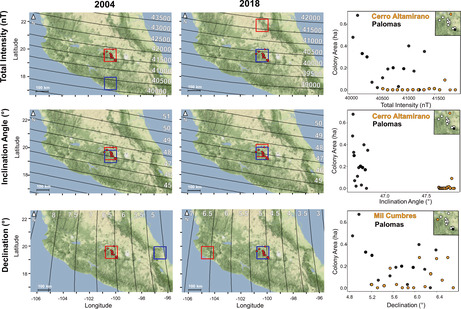

## 1 INTRODUCTION

Long‐distance animal migrants on their first journey face the daunting task of navigating and traveling to specific destinations without prior knowledge or experience. This problem is exacerbated for migrants that voyage on their own and that cannot rely on conspecifics that have previously completed the journey. One proposed mechanism facilitating migration for naïve migrants is via the use of a magnetic map, a set of instructions or cues that allows animals to navigate using parameters of the Earth's magnetic field (e.g., inclination angle, total intensity, declination; Chernetsov et al., 2017; Mouritsen, 2018; Putman, 2018). An inherited magnetic map provides migrants with information that allows them to know the direction that they need to travel and their position relative to the destination (Lohmann et al., 2008). Evidence for the use of a magnetic map imprinted as a juvenile for navigation has been demonstrated in marine migratory animals, such as hatchling sea turtles, juvenile salmon, and juvenile eels (Putman, 2018). In addition, exposure to specific geomagnetic cues along the migratory journey can trigger migration‐appropriate responses in inexperienced or naïve juvenile migratory birds (e.g., extension of fat deposition period – Fransson et al., 2001, changes in the amount of migratory restlessness – Bulte et al., 2017). Despite these findings, the use of imprinted or inherited geomagnetic map cues by other migratory animals, or the triggering effect of specific geomagnetic cues on migration, remains unknown.

Naïve fall monarch butterflies (*Danaus plexippus*) in Eastern North America potentially use geomagnetic map cues to migrate to overwintering sites in Central Mexico (Guerra, 2020). During the fall, millions of Eastern monarchs that developed in Southern Canada and the Northern United States migrate to a few overwintering sites in mountain ranges in Central Mexico (Brower, 1995; Urquhart & Urquhart, 1976). It remains unclear how fall monarchs that have never been to these sites find the same overwintering grounds year after year, especially since they are typically three generations removed from monarchs that made the previous fall migration.

Fall monarchs use sensory‐based compass mechanisms to maintain a southward flight orientation during fall migration (Guerra, 2020). The dominant mechanism used by monarchs is a time‐compensated sun compass (Froy et al., 2003; Mouritsen & Frost, 2002; Perez et al., 1997). Monarchs use the sun as a visual cue to maintain a southward heading and their internal circadian clock to compensate for the sun's position in the sky throughout the day. On overcast days when the sun is unavailable, migrants employ an inclination‐based magnetic compass as a backup mechanism to maintain southward directionality based on the inclination angle of Earth's magnetic field (Guerra et al., 2014; Wan et al., 2021). We note that early studies investigating magnetic orientation in monarchs (e.g., Mouritsen & Frost, 2002) did not activate this system because monarchs were not provided with necessary UV light wavelengths (Guerra et al., 2014; Wan et al., 2021). The magnetic compass, in tandem with the predictable correlation between the inclination angle of the geomagnetic field and latitude, serves as a second directional mechanism for flying southward.

Although these compasses can be used for maintaining proper flight directionality, monarchs cannot use these mechanisms for recognizing, locating, or stopping at the overwintering sites, as they only allow monarchs to determine direction. However, it is possible that monarchs use magnetic inclination parameters in combination with other geomagnetic cues to determine their location and the direction to fly to reach their destination (Guerra, 2020; Mouritsen, 2018; Reppert & de Roode, 2018). The possibility that monarchs possess this type of map sense remains controversial (Mouritsen et al., 2013a, 2013b; Oberhauser et al., 2013) and the role of geomagnetic cues remains untested.

Researchers have used displacement trials to test for the use of geomagnetic map cues. Here, individuals are displaced to unfamiliar, geographical locations to determine if they adjust their behavior to correct for the displacement. This technique revealed that red‐spotted newts (Fischer et al., 2001), spiny lobsters (Boles & Lohmann, 2003), and birds (Wiltschko, 2017) use Earth's magnetic field for navigation. Alternatively, animals have been tested in simulated geomagnetic displacement experiments. These studies subject individuals to artificially generated magnetic fields of locations different from the testing site, and the behavior of individuals is monitored for the expression of predicted responses or any changes in behavior, for example, a change in orientation behavior relative to what is observed or expected at a control site which was used to show that migratory sea turtles use geomagnetic map clues (Lohmann et al., 2012) and that fall migratory monarchs use an inclination‐based magnetic compass to maintain proper southwards flight (Guerra et al., 2014). A similar method for testing the existence of a geomagnetic map sense is to examine the behavior of animals in response to the Earth's shifting magnetic field over time, that is, secular variation of the geomagnetic field. This approach examines the behavior of individuals in response to the natural displacement of the Earth's magnetic field under natural conditions over time, and was used to show how juvenile salmon and sea turtles imprint geomagnetic cues at their birth site which are used to relocate the site when they return to breed (Lohmann et al., 2008; Putman & Lohmann, 2008).

**FIGURE 1:**
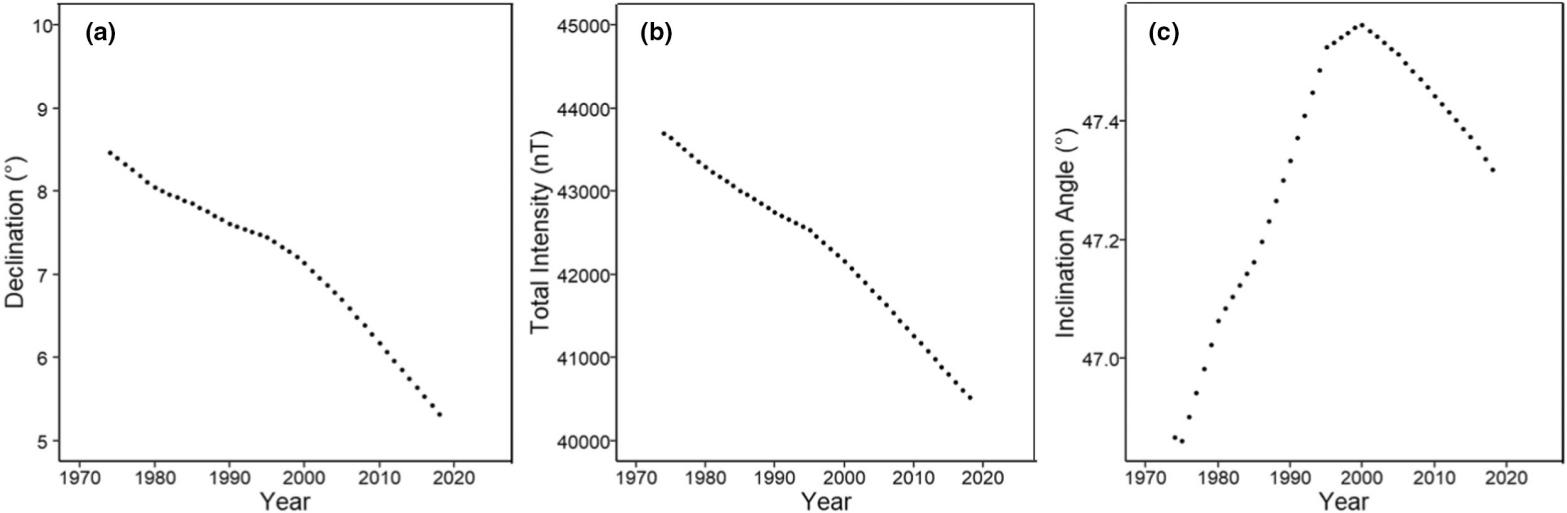
Change in geomagnetic parameters over time for (a) declination angle, (b) total intensity, and (c) inclination angle from 1974 until 2018 during November at a single overwintering site (19.850°N, 100.789°W). From 2004 to 2018, all geomagnetic parameters have a negative relationship as a function of time, indicating that monarch abundance should be decreasing at the more southern and/or eastern sites. The expectation is that if the butterflies are using the geomagnetic field associated with the geographical location of overwintering sites as either magnetic map sense guideposts or as “homing beacon” cues, we should see the strongest decline in abundance for the three most southerly sites. We note that inclination angle is cyclic, but during the monitoring period from 2004 to 2018 it was consistently declining.

We used a natural displacement approach to test the hypothesis that fall monarchs use geomagnetic cues to locate their overwintering sites in Central Mexico. Our study is the first to test if the choice of overwintering sites is correlated with geomagnetic cues possibly used to locate sites via a geomagnetic map sense navigational mechanism. We predict that if monarchs navigate to specific locations based on recognizing overwintering locations via long‐term magnetic map cues, there should be a shift in their overwintering range commensurate with the shift in the geomagnetic field (Figure 1). Due to the natural displacement of geomagnetic parameters from shifts in the geomagnetic field, we hypothesize that monarchs should adjust where they form overwintering aggregations, as evidenced by changes in colony size.

## 2 METHODS

We used data on the areal extent of overwintering colonies in Mexico collected by the World Wildlife Foundation funded Biosfera Mariposa Monarcha each December since 2004 to estimate colony abundance. Workers used a GPS device and walked the perimeter of forest encompassing each colony to determine the area of each colony. Subsequently, the GPS track was converted into a shapefile to calculate the area occupied by monarchs with GIS software (ArcGIS v3.3). The total area (ha) is used as an estimate of relative yearly abundance (Calvert & Brower, 1986; Slayback et al., 2007; Vidal et al., 2014; Vidal & Rendón‐Salinas, 2014). While newer sites have been located recently (Perez‐Miranda et al., 2020; Rendón‐Salinas et al., 2019a, 2019b; Vidal & Rendón‐Salinas, 2014), we examined data from 2004 to 2018 for 12 sites that have been consistently sampled every year since 2004: Sierra El Campanario, Cerro Altamirano, Palomas, San Francisco Oxtotilpan, Piedra Herrada, San Andres, Mil Cumbres, Sierra Chincua, Lomas de Aparicio, Las Palomas La Mesa, Sierra Chivati‐Huacal, and Cerro Pelon (Rendón‐Salinas & Galindo‐Leal, 2004; Rendón‐Salinas et al., 2005, 2006, 2007, 2008, 2009, 2011, 2014, 2015, 2016, 2017, 2018; Rendón‐Salinas et al., 2010; Rendón‐Salinas & Tavera‐Alonso, 2012, 2013). We used the earliest estimate in cases where butterflies were sampled multiple times in one year. We note that there is imprecision in these data as estimates of abundance, but the data are comparable due to similar methodology followed by workers and can be used to track change in abundance over time, which is the focus for this study. Moreover, as the data for these 12 sites were consistently sampled each year, we have an accurate measure of change in abundance at each site as a function of both time and the shift of the geomagnetic field from 2004–2018.

We calculated the geomagnetic field at each site based on the International Geomagnetic Reference Field (IGRF‐12), which provides historical data since 1900 based on date, latitude, and longitude (Thébault et al., 2015). We calculated the geomagnetic field for each site on November 15 of each year from 2004 to 2018. This date corresponds to the midpoint of the arrival of migrants, with monarchs typically beginning to arrive at the overwintering sites around November 1 (the Day of the Dead celebrations; Reppert & de Roode, 2018).

We calculated the geomagnetic field at each site, each year. We related each component of the geomagnetic field to the area occupied by overwintering butterflies (relative abundance) via a general linear model (glm) in R (R Core Team, 2021). The expectation is that if monarchs use the geomagnetic field to locate specific overwintering sites in Mexico, there would be a change in abundance at these sites related to the changing geomagnetic field. As migratory animals can use different parameters of the Earth's magnetic field, that is, inclination angle, total intensity, and magnetic declination, we examined each of these three geomagnetic parameters separately in our analyses.

## 3 RESULTS

**FIGURE 2:**
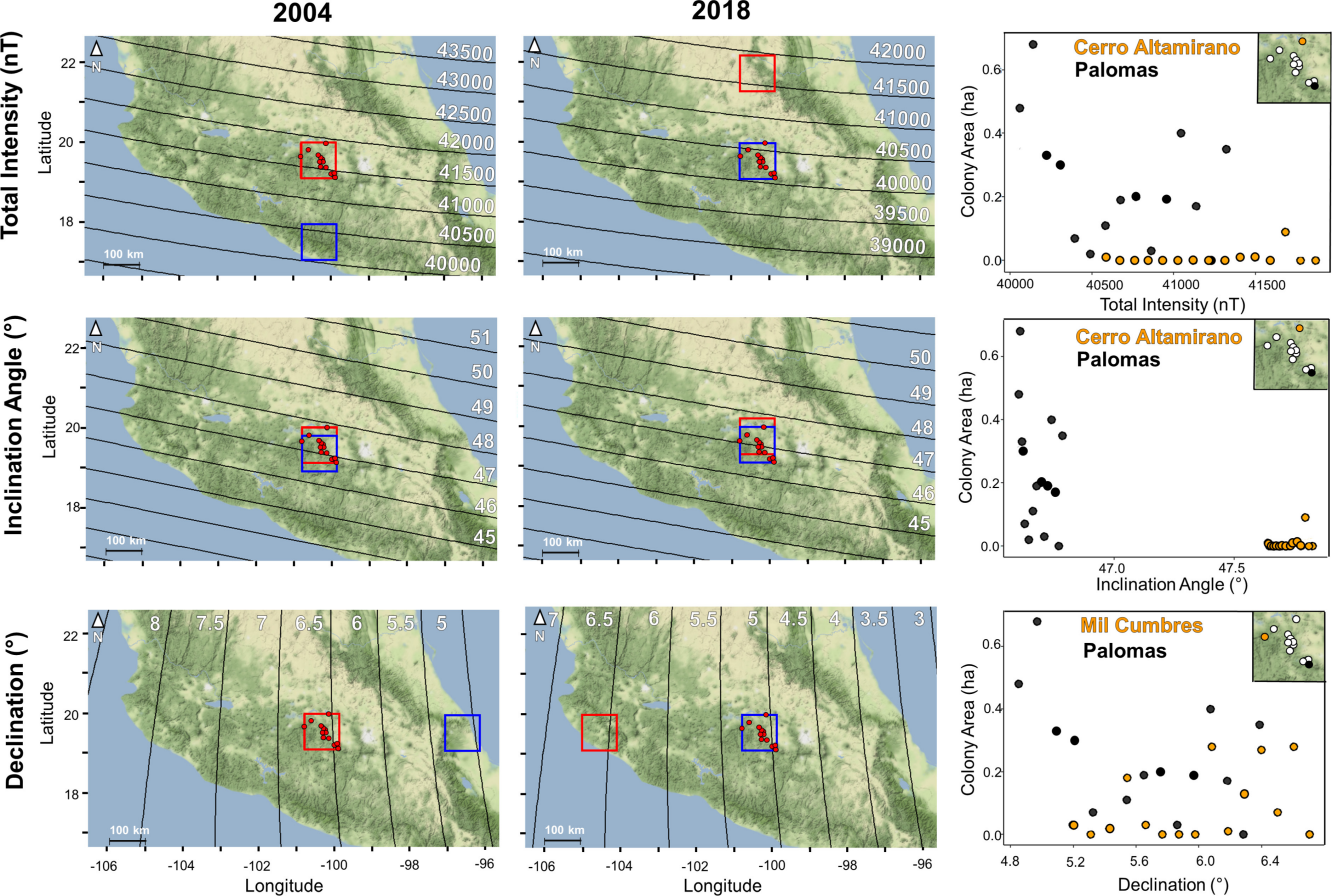
The location of monarch butterfly overwintering sites in Central Mexico with isoclinic lines in 2004 (left panel) and 2018 (middle panel) showing the shift in magnetic field. The red dots indicate the location of overwintering sites. For both the 2004 and 2018 maps, the red bounding box shows the overwintering site relative to total intensity (top row), inclination angle (middle row), and declination angle (bottom row) in 2004; the blue bounding box shows the same for 2018. For total intensity and declination angle, all overwintering sites fall outside of the displacement area due to the shift of the Earth's magnetic field. When considering inclination angle, the three most southern sites fall outside of the displacement area based on the shift in the Earth's magnetic field over this 14‐year period, yet monarchs still overwinter with similar abundances at these locations. The right panel represents overwintering sites on opposite ends of the natural displacement, either north–south (i.e., total intensity and inclination angle) or east–west (i.e., declination). For all three geomagnetic cues across all sites, there were no significant relationships between area occupied and total intensity, inclination angle, or declination. The black and orange dots correspond to the colony area (ha) as a function of the geomagnetic parameter at each overwintering site in the corresponding color and insert box of all overwintering sites.

**FIGURE 3:**
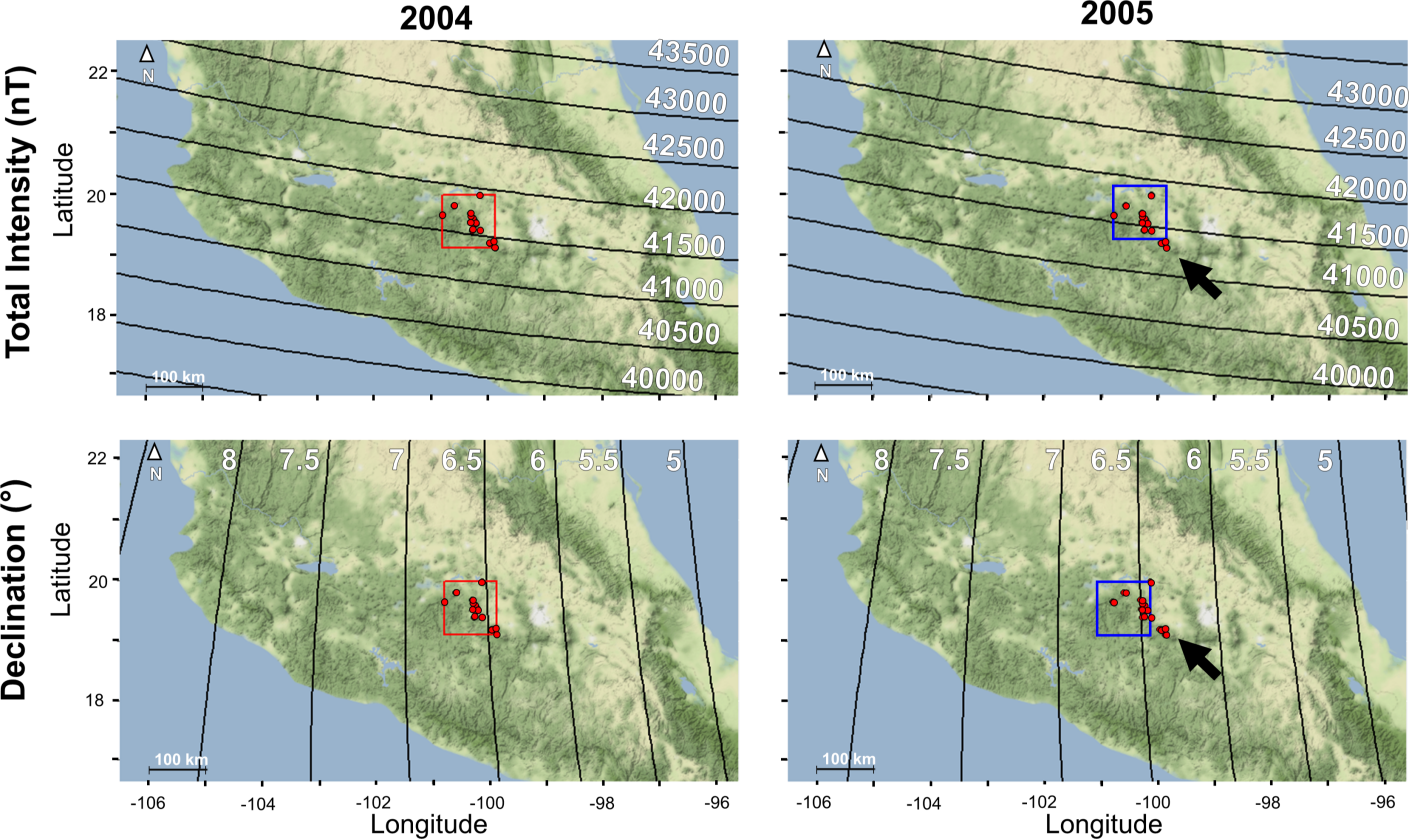
Change in total intensity (nT, top row) and declination angle (°, bottom row) over a one‐year interval from 2004 (left side, red box) to 2005 (right side, blue box) for the two geomagnetic parameters that had the greatest change over the 14‐year monitoring period. The red dots indicate the location of overwintering sites. Given the northward and westward shift, the southernmost sites (black arrow) would not be within the detectable region based on the geomagnetic parameters.

From 2004 to 2018, for each of the 12 overwintering sites in Central Mexico that we examined, all 3 geomagnetic parameters examined consistently shifted (Figure 1). The total intensity of the geomagnetic field decreased by an average of 1264 ± 1.519 nT. The magnitude of decrease in total intensity was equivalent to a northward displacement of 140 km (Figure 2) or 10 km/year (Figure 3). Similarly, magnetic inclination values decreased by an average of 0.173 ± 0.003°. The magnitude change in inclination angle over this time was equivalent to moving 30 km northward (Figure 2) or 2.1 km/year. Magnetic declination values decreased by an average of 1.529 ± 0.002°, equal to a westward geographic displacement of 300 km (Figure 2) or 21.4 km/year (Figure 3). Shifts in total intensity and declination should have moved all overwintering sites outside of the historical range, while changes in inclination would have shifted the three most southern overwintering sites out of the historical overwintering range (Figure 2). Individual geomagnetic parameters indicate that overwintering sites would have been geographically displaced northward (total intensity and inclination angle; Figure 2) or westwards (declination angle; Figure 2). If geomagnetic parameters were used as part of a bicoordinate map signature (e.g., total intensity and inclination angle), there would be significant discordance between these parameters in how far and where each overwintering site has shifted.

**FIGURE 4:**
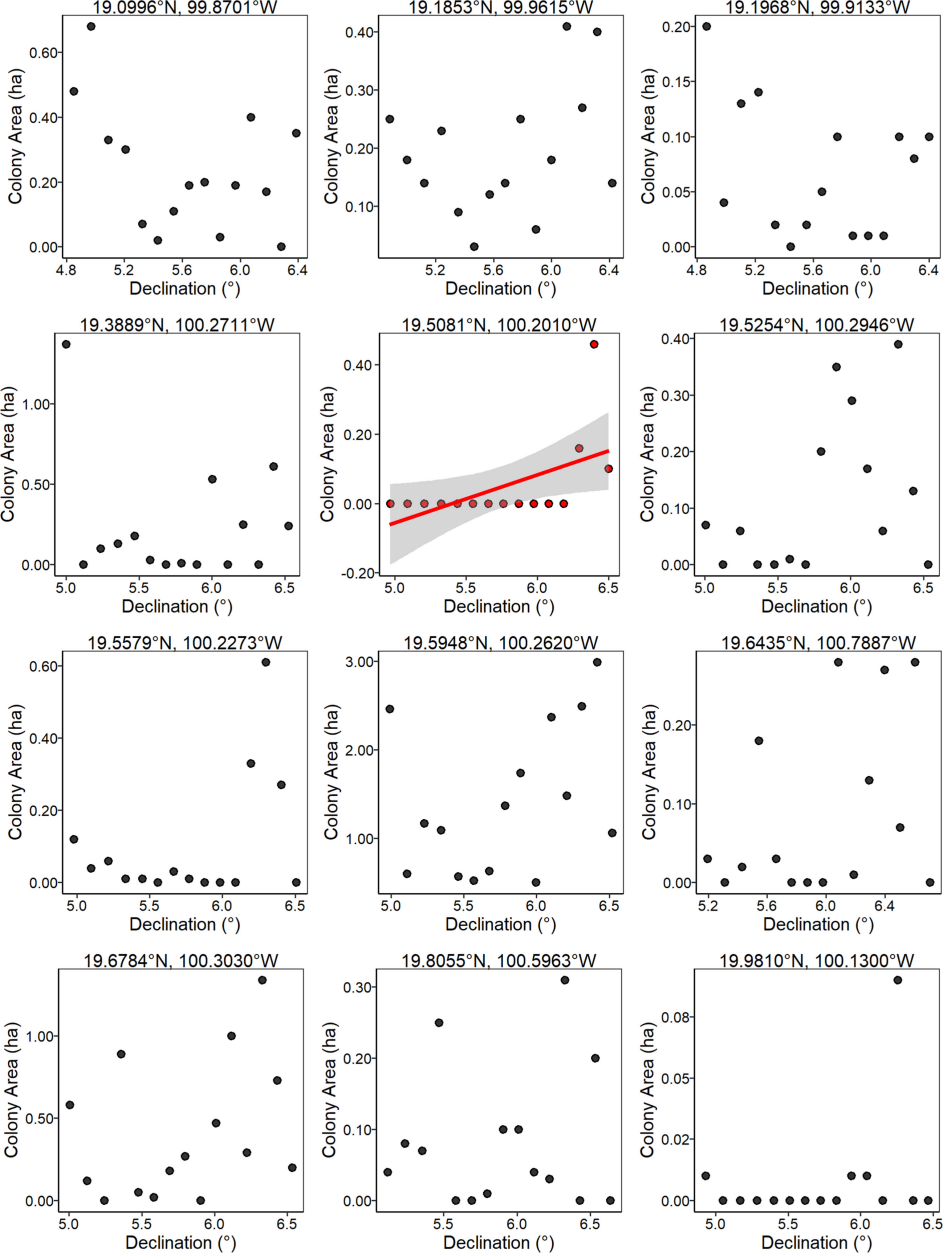
The relationship between overwintering colony size of *D. plexippus* (ha) and the declination angle of the geomagnetic field for 12 overwintering sites with data from 2004 to 2018. Sites are ordered from south to north, with the most southern site first. There was no relationship between colony size and magnetic declination for 11 of these sites. The significant relationship for Sierra El Campanario (*p* = .035, *r* ^2^ = .24; trend line in red with 95% confidence intervals) should be viewed with caution, as it violates the homoscedasticity assumption for linear regression and represents extreme observations where since 2007 monarchs were not found at this site. Note that the scales differ among plots for colony area (ha) and declination.

**FIGURE 5:**
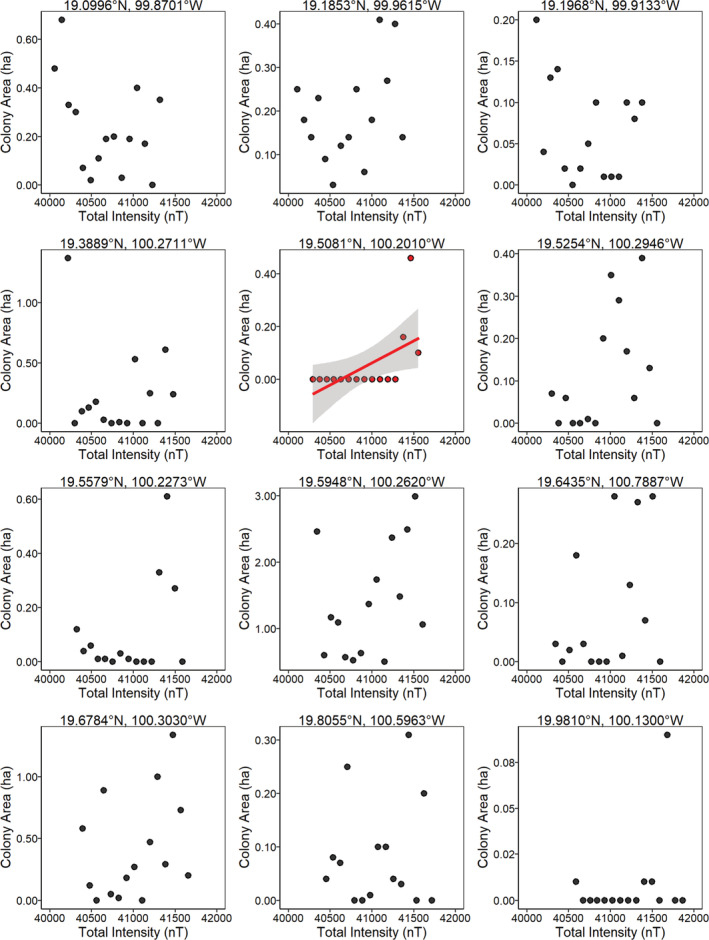
The relationship between overwintering colony size of *D. plexippus* (ha) and the total intensity (nT) of the geomagnetic field for 12 overwintering sites with data from 2004 to 2018. Sites are ordered from south to north, with the most southern site first. There was no relationship between colony size and total intensity for 11 of these sites. The significant relationship for Sierra El Campanario (*p* = .031, *r* ^2^ = .26; trend line in red with 95% confidence intervals) should be viewed with caution, as it violates the homoscedasticity assumption for linear regression and represents extreme observations where since 2007 monarchs were not found at this site. Note that the scales differ among plots for colony area (ha).

**FIGURE 6:**
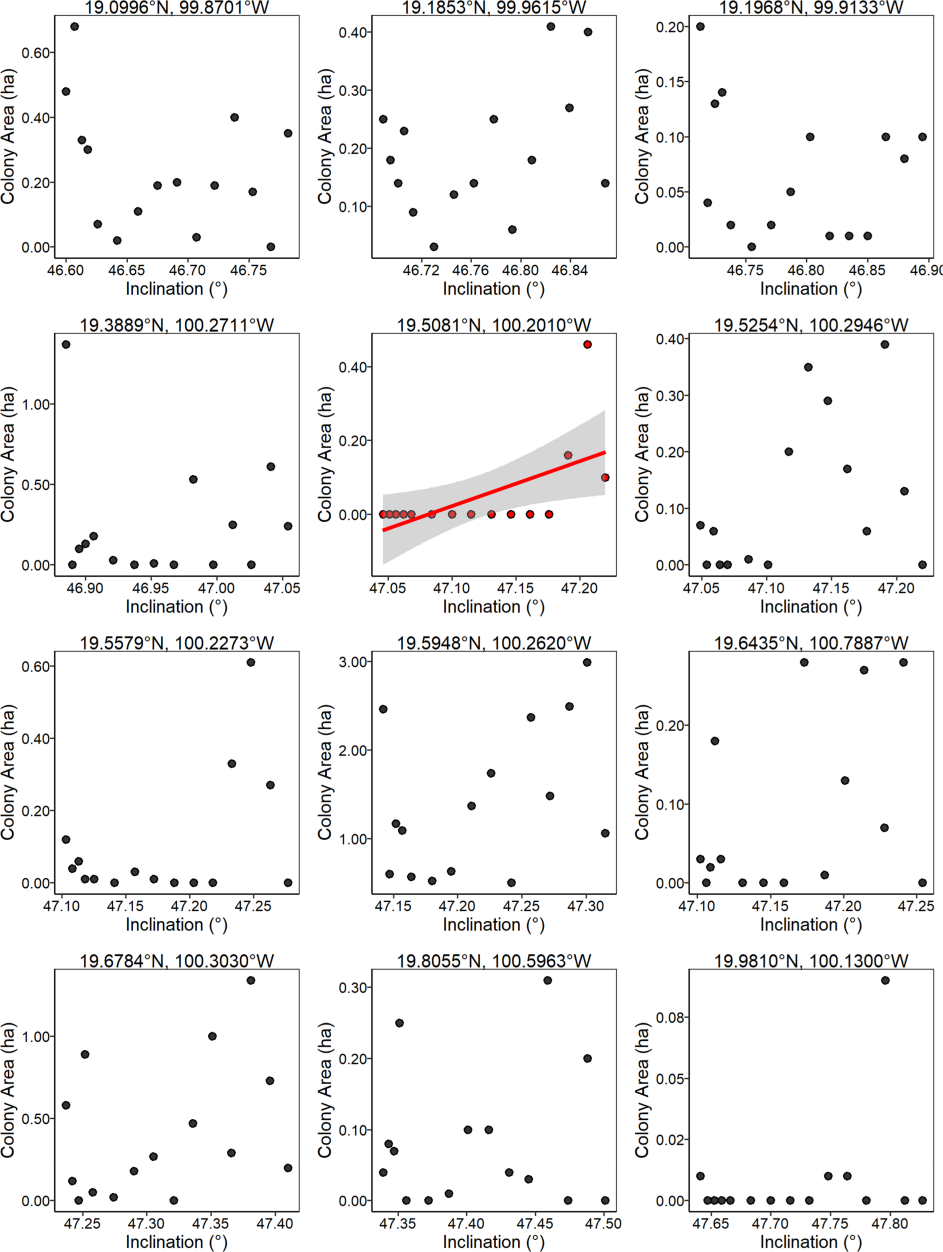
The relationship between overwintering colony size of *D. plexippus* (ha) and the inclination angle of the geomagnetic field for 12 overwintering sites with data from 2004 to 2018. Sites are ordered from south to north, with the most southern site first. There was no relationship between colony size and magnetic inclination for 11 of these sites. The significant relationship for Sierra El Campanario (*p* = .022, *r* ^2^ = .29; trend line in red with 95% confidence intervals) should be viewed with caution, as it violates the homoscedasticity assumption for linear regression and represents extreme observations where since 2007 monarchs were not found at this site. The three most southerly sites had no significant relationships with colony area as a function of inclination angle. Note that the scales differ among plots for colony area (ha) and inclination.

**FIGURE 7:**
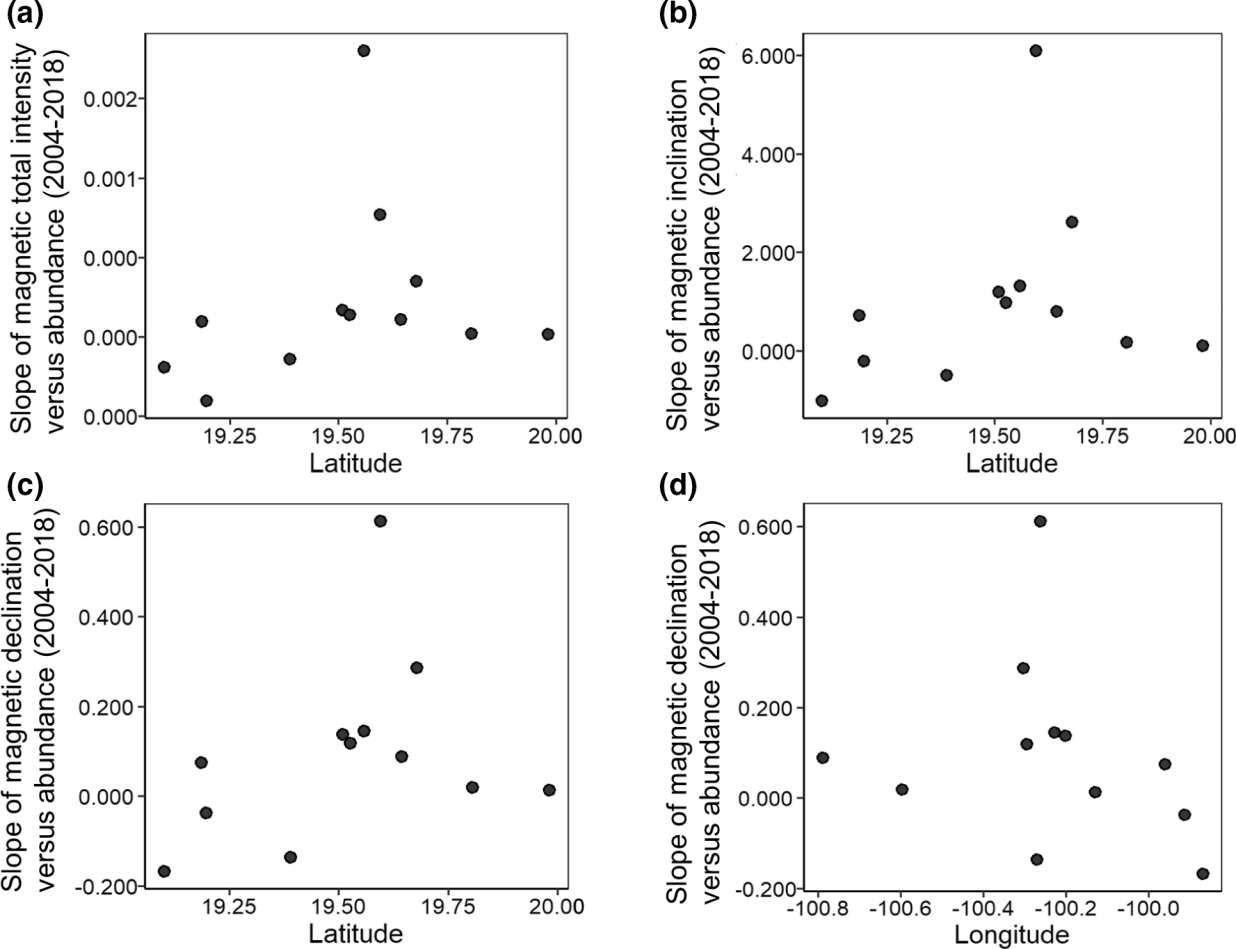
No relationship between the slope of colony size and geomagnetic (a) total intensity, (b) inclination angle, and (c) declination angle versus the latitude of the overwintering sites (black dot) was found. There was also no relationship between the slope of colony size and geomagnetic (d) declination versus the longitude of the overwintering sites. If monarchs were responding to the changing geomagnetic field, we would expect more southerly sites to have greater slopes (decreasing abundance) relative to more northerly sites. Thus, there should be a positive slope in the relationship shown here; however, the slope was not significantly different than zero for total intensity (*β* = 0.00055 ± 0.00067, *t* = 0.83, *p* = .42), inclination angle (*β* = 2.00 ± 2.16, *t* = 0.93, *p* = .38), or declination angle (*β* = 0.26 ± 0.23, *t* = 1.14, *p* = .27). In the case of declination, we would expect more westerly sites to have greater slopes. Thus, we would expect a negative slope in the relationship; however, the slope was not significantly different than zero for declination based on longitude (*β* = −0.18, *t* = −0.76, *p* = .46).

If fall monarchs use parameters of the Earth's magnetic field at the overwintering sites as inherited cues for locating these sites, then monarch abundance at these sites should have declined over time and/or the sites would cease to be used for overwintering (Figure 1). However, we found no evidence that the use of these sites changed with changes in any parameter of the geomagnetic field (Figures 4, 5, 6), indicating that fall monarchs do not use consistent inherited geomagnetic map cues for locating overwintering sites in Mexico. Our analysis shows that monarchs do not alter their overwintering behavior, that is, roost formation, in response to geographical displacement, either northward or westward, of the geomagnetic parameters of the overwintering sites over time. In only one case (Lomas de Aparicio – 19.508°N, 100.201°W, Figures 4, 5, 6) was there a significant relationship between the estimated abundance of the overwintering colony and the decrease in magnetic inclination. This site has also had no butterflies since 2007; therefore, it was not well‐suited for analysis by linear regression. Across all sites, there was no trend for a south to north, nor an east to west, increase or decrease in abundance of overwintering monarchs that would be consistent with monarchs sensing and tracking the changes in the geomagnetic signatures of overwintering sites over time (Figure 7).

## 4 DISCUSSION

Given the large secular shift in the geomagnetic field and a lack of change in the abundance of monarchs at the 12 different overwintering sites that have been consistently monitored each year over time (2004–2018), there is no long‐term geomagnetic site specificity for monarch butterflies. The results from this natural displacement study are inconsistent with fall Eastern North American monarchs possessing a long‐term (i.e., relatively fixed) inherited innate magnetic map sense to locate the same overwintering sites in Mexico year after year (Figure 2). Over the past decade, researchers have searched and registered the presence of overwintering sites in other areas in Mexico to monitor the overwintering monarch population, especially any outside the typical overwintering area, for example, the Monarch Butterfly Biosphere Reserve (Perez‐Miranda et al., 2020). In contrast to tracking changes in the geomagnetic signature, all new sites that have been located are to the southeast of the typical overwintering area (Perez‐Miranda et al., 2020), in direct contrast to changes in the geomagnetic field.

The behavior of monarchs could be like the behavior of naïve individuals of other migratory species, for example, sea turtles and salmon, that use geomagnetic map signatures to locate sites during migration (Putman, 2018). These species use geomagnetic cues that are imprinted and calibrated at birth but are recalibrated to recent magnetic conditions. For monarchs, the magnetic signature would need to be environmentally cued and then epigenetically inherited, that is, “adjusted” each year, and inherited from those that reach and overwinter in Mexico the year prior to at least two subsequent generations. This mechanism could allow monarchs to overwinter at the same geographical sites each year, despite the annual change in the geomagnetic parameters of these locations due to the shift in the geomagnetic field. This type of magnetic map sense may be part of the monarch migratory syndrome, the same way that southward oriented directional flight, the hallmark trait of fall migrants, is part of the fall monarch migratory syndrome (Guerra, 2020). The monarch migratory syndrome is a polyphenic trait that is triggered by exposure to specific environmental conditions, for example, decreasing sun angle and photoperiod, as well as cooler and fluctuating temperatures that occur between late summer and fall (Goehring & Oberhauser, 2002).

This type of inherited, annually updated magnetic map mechanism could also involve the use of a very broad scale map sense (e.g., the intersection of an individual magnetic parameter, such as inclination angle or total intensity – Lohmann et al., 1999, bicoordinate map location based on inclination angle and total intensity – Putman et al., 2011, or differences in longitude via magnetic declination – Chernetsov et al., 2017), which could serve to indicate a general location of the overwintering sites, for example, a region or suitable habitat indicated by a geomagnetic cue, on a magnetic map. In contrast to sensing specific geomagnetic signatures (as above), this broad map sense would encompass a very large area. Here, locating the actual overwintering sites might then involve sensing other cues once near or inside this area, presumably close‐range cues, denoting the overwintering sites (Mouritsen, 2018).

Although the use of a magnetic map sense (whether to relatively specific or broad areas indicated by geomagnetic cues) potentially explains the capability of monarchs to find the same sites each year despite secular variation, several aspects of the monarch migration make these possibilities unlikely. It is unlikely that monarchs use yearly recalibrated, inherited geomagnetic map cues. Geomagnetic parameters (declination and total intensity) showed mean yearly shifts in different directions and magnitudes that would be sufficient to alter yearly overwintering abundance at the current overwintering sites (Figure 3). While bioclimatic models have shown new, potential regions of interest where monarchs have been recently found (Perez‐Miranda et al., 2020; Rendón‐Salinas et al., 2019a, 2019b; Vidal & Rendón‐Salinas, 2014), the fact that these potential sites are south of the change in geomagnetic parameters supports the lack of an inherited geomagnetic map as these parameters have been shifting northwards and westwards annually and in other directions over longer time periods.

It is also unlikely that fall monarchs possess an inherited large‐scale (100 s of km) magnetic map sense (Lohmann et al., 2001). If monarchs possessed an inherited large‐scale magnetic map sense, they would be expected to overwinter across a much wider geographical range (Figure 2). Oyamel firs, the primary species on which monarchs overwinter, exist well outside the current monarch overwintering range (Jaramillo‐Correa et al., 2008; Perez‐Miranda et al., 2020; Sáenz‐Romero et al., 2012), but monarchs also form roosts on cedar, pine, or oak trees in Mexico (Brower et al., 2008; Garcia‐Serrano et al., 2004), and moreover, during the journey south in the fall, Eastern monarchs roost on many species of trees, for example, maple, oak, pecan, willow, walnut, ash, elm, hackberry, and palm (Davis et al., 2012). Monarchs roosting on oyamel firs that can be found outside the current monarch overwintering range and on a diversity of trees besides oyamel firs suggest that monarchs should be able to use new locations indicated by shifting geomagnetic parameters, even at large scales. Monarchs, however, have not adjusted their selection of overwintering locations in Mexico nor has their abundance shifted from specific sites in concordance with the natural displacement of the Earth's magnetic field.

That fall western monarchs from Arizona can migrate to and overwinter in either Mexico or California (Billings, 2019; Morris et al., 2015) also argues against an inherited specific or large‐scale magnetic map sense. Monarchs caught, tagged, and released on the same day from the same location were found overwintering in either California or Mexico (Billings, 2019). Similarly, if monarchs possess an inherited magnetic map sense, there should also be genetic differentiation between Eastern and Western monarchs; however, Eastern and Western monarchs are genetically identical (Freedman et al., 2021). The patterns and observations found in our study provide compelling evidence that indicates that monarchs do not use genetically inherited geomagnetic map cues for migrating to and finding overwintering sites. Our results therefore answer a long‐standing question in the migratory biology of monarchs and provide further insight into the broader question of the potential for geomagnetic map sense navigation in animals outside of species for which this has been studied.

How then do naïve fall Eastern North American migratory monarchs, who have never been to their destination, locate overwintering sites each year? It is likely that monarchs use their compass mechanisms (e.g., time‐compensated sun compass and inclination‐based magnetic compass) to maintain a southward flight heading during migration until they reach the border between the United States and Mexico. They may then use the geography of Mexico (e.g., the mountains to the West and the Gulf of Mexico to the East) to get funneled to their overwintering sites while continuing to fly in a southerly direction (Calvert, 2001; Mouritsen, 2018). Once near the overwintering sites, monarchs may then use strategies in which they use short‐range or local cues, respectively, for determining overwintering sites (Fischer et al., 2001; Mouritsen, 2018). Monarchs might also use olfactory cues, for example, cues left by monarchs from past migrations or volatiles from trees that monarchs overwinter on (Mouritsen, 2018; Reppert & de Roode, 2018).

One key possibility is that monarchs might recognize and locate their overwintering sites via habitat selection, as they may be looking for specific microclimates while flying south, which are provided by these overwintering areas. An important aspect of the microclimate at overwintering sites is that it provides temperatures that are cold enough to keep metabolic demands low during overwintering, produce cold conditions that can recalibrate the time‐compensated sun compass for northward oriented flight during the spring remigration (Guerra & Reppert, 2013), but do not cause freezing (Brower et al., 2008, 2009). Monarchs might therefore also use temperature cues as part of microclimate selection to locate these sites. Evidence supporting this is that the overwintering sites in Mexico and California share similar temperature conditions during the period in which monarchs overwinter (Guerra & Reppert, 2013), whereas these sites are significantly different in geomagnetic field parameters, tree species used for overwintering (e.g., oyamel fir forests in Mexico and Eucalyptus trees, Monterey pines, and Monterey cypresses in California), environmental conditions (e.g., high altitude mountainous forests in Mexico and areas close to sea level in California), and level of human activity (e.g., urbanized versus rural areas). Once fall migratory monarchs reach these key microclimates, regardless of whether they are in Mexico or California, such temperature conditions, potentially in conjunction with other environmental cues that coincide with their arrival in these conditions (e.g., the loss or the lack of a specific solar angle that triggers southward directional flight in fall migrants; Parlin et al., 2022), might then trigger other aspects of the migratory biology of monarchs that then keep them there for the entire overwintering period. That fall monarchs have not been observed significantly south of the overwintering sites during the overwintering period supports this possibility.

## AUTHOR CONTRIBUTIONS

**Patrick A. Guerra:** Conceptualization (equal); writing – review and editing (equal). **Adam F. Parlin:** Formal analysis (supporting); visualization (lead); writing – review and editing (equal). **Stephen F. Matter:** Conceptualization (equal); formal analysis (equal); visualization (supporting); writing – original draft (lead); writing – review and editing (equal).

## CONFLICT OF INTEREST

The authors declare no conflicts of interest.

## Data Availability

The data used in this study are publicly available. Our compiled data is open access available on the University of Cincinnati's Scholar@UC digital repository https://doi.org/10.7945/74qa‐e577.
